# Recruiting Racially and Ethnically Diverse Dementia Family Caregivers for Survey Research

**DOI:** 10.1093/geroni/igaa057.1135

**Published:** 2020-12-16

**Authors:** Kyra Mendez, Hae-Ra Han

**Affiliations:** Johns Hopkins University School of Nursing, Baltimore, Maryland, United States

## Abstract

Recruiting racially and ethnically diverse dementia family caregivers (FCGs) for research can be challenging. The purposes of this presentation are to describe methods of successfully recruiting racially and ethnically diverse dementia FCGs for survey research and to share lessons learned. This study aimed to recruit dementia FCGs who have a chronic health condition and access to a mobile device. FCGs were primarily recruited from the Baltimore-Washington Metropolitan area to complete an online or phone survey about their technology use. A variety of recruitment methods were used including: posting ads in a local newspaper targeting older adults, partnering with a local Alzheimer’s research center and memory treatment center, attending community events, using online research registries, and posting online advertisements. The most successful method of recruiting minorities was by attending community events for caregivers and talking directly with community members about the research study. Online recruitment methods were less successful, but yielded the greatest diversity of participants, including Asian, Native American, mixed race, and African American FCGs. Some challenges associated with recruiting minority FCGs were working with primarily immigrant communities; recruiting FCGs who do not speak English; and building trust among communities that have been negatively impacted by research. Suggestions for future research include using recruitment strategies that enable researchers to build rapport with FCGs, engaging community stakeholders to understand your source population, and using a variety of recruitment methods. Also, online recruiting through credible sources appears to be a somewhat feasible method of recruiting diverse FCGs for survey research.

